# Novel Mutations in K13 Propeller Gene of Artemisinin-Resistant *Plasmodium falciparum*

**DOI:** 10.3201/eid2103.140898

**Published:** 2015-03

**Authors:** Rie Isozumi, Haruki Uemura, Isao Kimata, Yoshio Ichinose, John Logedi, Ahmeddin H. Omar, Akira Kaneko

**Affiliations:** Osaka City University, Osaka, Japan (R. Isozumi, I. Kimata, A. Kaneko);; Institute of Tropical Medicine, Nagasaki University, Nagasaki, Japan (H. Uemura, Y. Ichinose);; Ministry of Public Health and Sanitation, Nairobi, Kenya (J. Logedi, A.H. Omar);; Karolinska Institutet, Stockholm, Sweden (A. Kaneko)

**Keywords:** Plasmodium falciparum, artemisinin, drug resistance, point mutation, parasites, malaria, Kenya, parasites, antimicrobial resistance

## Abstract

We looked for mutations in the *Plasmodium falciparum* K13 propeller gene of an artemisinin-resistant parasite on islands in Lake Victoria, Kenya, where transmission in 2012–2013 was high. The 4 new types of nonsynonymous, and 5 of synonymous, mutations we detected among 539 samples analyzed provide clues to understanding artemisinin-resistant parasites.

The worldwide spread of malaria parasites with resistance to antimalarial drugs has been a serious concern over the past few decades. During the 2000s, *Plasmodium falciparum* parasites acquired resistance to key drugs, such as chloroquine and sulfadoxine–pyrimethamine, in many malaria-endemic countries, including Kenya (*1*). Artemisinin-based combination therapy (ACT) has been introduced in most malaria-endemic countries and is the first-line therapy. However, the first clinical cases of artemisinin resistance in western Cambodia were reported in 2008 (*2*), and *P. falciparum* with reduced in vivo susceptibility to artesunate in western Cambodia was reported in 2009 (*3*,*4*). On the basis of these findings, genome-wide analyses of artemisinin-resistant *P. falciparum* isolates found strong correlations between a mutant allele in the K13 propeller, in vitro parasite survival rates, and in vivo parasite clearance rates; these correlations indicate that mutations in the K13 propeller (especially C580Y, R539T, and Y439H) are important determinants of artemisinin resistance (*5*,*6*). Analysis of parasites from several Cambodian provinces indicated that K13 propeller mutations are rarely observed in samples from provinces without documented resistance but are prevalent in provinces with reported resistance (*6*,*7*).

In Kenya, ACT was introduced as first-line therapy for *P. falciparum* malaria in 2004 (*1*). However, various types of antimalarial drugs—including chroloquine, sulfadoxine–pyrimethamine, and artemether/lumefantrine—are available for purchase at pharmacies without physicians’ prescriptions. In this study, we describe some K13 propeller mutations of *P. falciparum* parasites from western Kenya.

## The Study

The research was conducted at 4 islands on Lake Victoria (Kibuogi, Ngodhe, Takawiri, Mfangano) and 1 shoreline community of Mbita District (Ungoye) in western Kenya (Figure). In this area, the PfPR_2–10_ (community *P. falciparum* parasite rate standardized to the 2- to 10-year age group) was reported in 2009 to be >40% (*8*). Although in some area of Kenya malaria has decreased, its prevalence remains high in the Lake Victoria basin because of the lake environment (*8*–*10*). In 2009, a total of 50%–70% of households owned insecticide-treated bed nets (*11*), which substantially reduce the risk for transmission of malaria parasites by providing barriers against mosquitoes. Although malaria is annually more prevalent in the 2 wet seasons (March–June and October–November) (*9*), in the study sites, it is highly endemic throughout the year.

**Figure:**
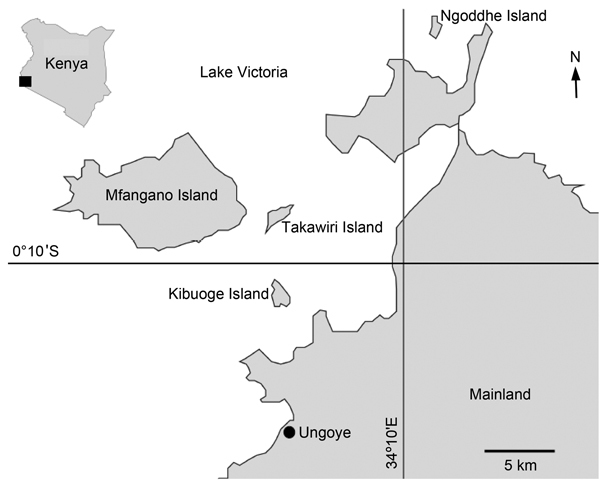
Study sites for investigation of K13 propeller gene in *Plasmodium falciparum*, Mbita District, Kenya, 2012–2013. Insert shows location of study area in Kenya.

Filter paper blood spots were collected from participants (Technical Appendix Table 1) during population-wide cross-sectional malaria surveys conducted in February and August 2012 and August 2013 at the 5 study sites. We obtained ethics approval from the Kenyatta National Hospital and the University of Nairobi. All study participants provided informed consent.

Parasitic DNA was extracted from the filter paper (*12*), and *P. falciparum* DNA was detected by a mitochondrial DNA-based PCR (*13*). Sequencing of the K13 propeller gene was attempted on the diagnostic PCR-positive specimens (online Technical Appendix). The prevalence of *P. falciparum*, as determined by PCR, in the rainy and dry seasons was 7.2%–26.2% and 6.5%–15.5% on the 3 small islands (Kibuogi, Ngodhe, and Takawiri), 47.3% and 31.4% on Mfangano Island, and 38.4% and 41.9%–64.0% in Ungoye, respectively (Table 1).

**Table 1:** Prevalence of *Plasmodium falciparum* and analysis of K13 propeller gene, Mbita District, Kenya, 2012–2013

Time	Study site	Total no. samples	PCR positive, no (%)*	K13 propeller gene analyzed, no.
2012 Feb	Kibuogi	130	34 (26.2)	21
	Ngodhe	250	18 (7.2)	5
	Takawiri	250	34 (13.6)	19
	Mfangano	427	202 (47.3)	138
	Ungoye	250	96 (38.4)	69
2012 Aug	Kibuogi	195	17 (8.7)	10
	Ngodhe	232	36 (15.5)	18
	Takawiri	230	15 (6.5)	9
	Mfangano	706	222 (31.4)	145
	Ungoye	248	104 (41.9)	65
2013 Aug	Ungoye	250	160 (64)	40

*PCR detected *Plasmodium falciparum* only.

We successfully analyzed 539 samples for the K13 propeller gene. Nine new types of point mutations were identified among these samples (Table 2). Participants infected with parasites harboring a mutation on the K13 propeller gene are listed in Technical Appendix Table 2. The sequences reported in this study have been deposited in the DDBJ/EMBL/GenBank databases (accession nos.AB936059–AB936067).

**Table 2:** Observed mutations in the K13 propeller gene in *Plasmodium falciparum,* Mbita District, Kenya, 2012–2013

Mutation	Amino acid change and location	Genetic change	Study site (no. isolates)		
			2012 Feb	2012 Aug	2013 Aug
Nonsynonymous	M442V	ATG →GTG		Mfangano (1)	
	N554S	AAT →AGT	Ungoye (1)		
	A569S	GCA →TCA			Ungoye (1)
	A578S	GCT →TCT	Mfangano (4)	Mfangano (1)	
Synonymous	C439C	TGC→TGT	Ungoye (2)		
	S477S	TCT →TCG	Takawiri (1)		
	Y500Y	TAT→TAC		Mfangano (1)	
	N531N	AAT →AAC			Ungoye (1)
	G538G	GGT→GGA	Mfangano (3)		

## Conclusions

We detected 4 novel nonsynonymous and 5 novel synonymous mutations in the highly conserved K13 propeller gene of *P. falciparum* parasites from western Kenya. Ariey et al. noted that the frequency of mutant alleles strongly correlated with the prevalence of day 3 positivity after ACT treatment in humans in Cambodia and that those mutations reflected positive selection (*6*). That study found 17 mutant alleles in the K13 propeller gene. Among them, C580Y, R539T, and Y493H were prevalent and strongly related to in vivo delayed parasite clearance. In our study, all the mutations found differed from those reported in Cambodia, and mutant alleles were not always observed in the proceeding seasons, so some mutations could be occasionally introduced. Most of those mutations are not suitable for the life cycle of parasites, and only a few suitable for survival under the conditions of artemisinin selection pressure could be selected.

We observed no identical mutations at >2 of these 5 study sites. Furthermore, only 1 type of mutation, A578S from Mfangano Island, was detected during 2 seasons, whereas other mutations were not observed in the next season, half a year later. Any family relations were not identified among the 4 participants harboring A578S mutation in February 2012 at Mfangano Island. Point mutations can occasionally occur on the *P. falciparum* K13 propeller gene as a standing variation, but most of the isolates that recently acquired the mutation may disappear because of some fitness disadvantage or the effect of a random genetic drift (*14*).

Malaria parasites grow and multiply at 2 different biologic stages in humans and mosquitoes. Therefore, isolates with new mutations must adapt to both circumstances. We detected the mutant allele A578S in the K13 propeller gene in 2 consecutive seasons on Mfangano Island. This mutation, which modifies amino acids from being hydrophobic to hydrophilic, is close to the prevalent single nucleotide polymorphism C580Y from Cambodia that is thought to be necessary in protein–protein interactions, which could affect artemisinin susceptibility. The genotype analyses of the parasites from this island are critical to understanding the role of this mutation and ACT efficiency in this geographic area.

Our K13 propeller sequence analysis of *P. falciparum* parasites from a malaria-endemic area in Kenya did not detect the predicted artemisinin-resistant genotypes, but we observed some temporal substitutions. A limitation of our study was that the sample size was insufficient to specifically provide an understanding of this result. The accumulation of data from this region and from other malaria-endemic areas will increase our understanding of the relationship between the K13 propeller gene and artemisinin resistance. Monitoring these molecular markers and the efficacy of antimalarial drugs is critical for increasing understanding of artemisinin resistance and predicting its spread. This study identified clues that are essential in understanding artemisinin-resistant parasites.

Technical Appendix Demographic information about the study participants; sequencing of the *Plasmodium falciparum* K13 propeller gene; and data on the participants who had parasites harboring a mutation on the K13 propeller gene.
