# Supplementary material for: Novel Mutations in K13 Propeller Gene of Artemisinin-Resistant Plasmodium falciparum
Source: Emerg Infect Dis. 2015 Mar;21(3):490–2. doi: 10.3201/eid2103.140898 (PMC4344268; doi:10.3201/eid2103.140898)
Supplement: Technical Appendix — Demographic information about the study participants; sequencing of the Plasmodium falciparum K13 propeller gene; and data on the participants who had parasites harboring a mutation on the K13 propeller gene. [file 14-0898-Techapp-s1.pdf]

Article DOI: http://dx.doi.org/10.3201/eid2103.140898

Novel Mutations in K13 Propeller Gene of Artemisinin-Resistant *Plasmodium falciparum*

Technical Appendix

Technical Appendix Table 1. Demographic information of the study participants

Time	Study site	Total, no.	Age, y*	Sex, no. M/F
2012 Feb	Kibuogi	130	14.1 ± 13.9 (0–67)	48/82
	Ngodhe	250	18.2 ± 16.0 (0–70)	106/144
	Takawiri	250	18.2 ± 15.9 (0–81)	106/143
	Mfangano	427	16.2 ± 16.1 (0–80)	191/236
	Ungoye	250	19.2 ± 17.4 (0–80)	119/131
2012 Aug	Kibuogi	195	16.1 ± 15.4 (0–85)	88/107
	Ngodhe	232	16.1 ± 14.5 (0–80)	115/117
	Takawiri	230	16.0 ± 17.4 (0–69)	109/121
	Mfangano	706	19.6 ± 18.4 (0–80)	344/363
	Ungoye	248	18.1 ± 15.6 (0–79)	121/127
2013 Aug	Ungoye	250	18.9 ± 17.3 (0–88)	112/138

*Average age ± SD (age range).

Sequencing of the *P. falciparum* K13 Propeller Gene

The K13 propeller domain was amplified by nested PCR using the following primers: for the primary PCR (kelch-out-f 5′-gggaatctggtggtaacagc-3′ and kelch-out-r 5′-cggagtgaccaaatctggga-3′) and the nested PCR (kelch-in-f 5′-gccttgttgaaagaagcaga-3′ and kelch-in-r 5′-gccaagctgccattcatttg-3′). Nested PCR product was 849 bp and corresponding to nt 1279–2127 (representing codons 427–709) of PF3D7_1343700 K13 propeller domain, which included mutations related to delayed parasite clearance (*1*). In the 20-µL first-round reaction, 2 µL of DNA was amplified with 500 nM of each primer, 10 µL of GoTaq Green Master Mix (Promega, Madison, WI, USA). Cycling conditions were 95°C for 1 min, followed by 35 cycles at 95°C for 20 sec, 57°C for 20 sec, and 60°C for 150 sec, with an extension at 60°C for 3 min. For the secondary PCR, 2 µL of 100× diluted first PCR products were used for template. In the 20-µL second-round reaction, 2 µL of DNA template was amplified with 500 nM of each primer, 10 µL of GoTaq Green Master Mix. Cycling conditions were 95°C for 1 min, followed by 35 cycles at 95°C for 20 sec, 55°C for 20 sec, and 60°C for 1 min, with an extension at 60°C for 3 min. Secondary PCR products were purified by ExoSAP-IT (Affymetrix, Santa Clara, CA, USA) and sequenced by using BigDye Teminator v.1.1 (Life Technologies, Carlsbad, CA, USA) according

to manufacturer's instruction. The primers for sequencing were same with those of nested PCR (kelch-in-f and kelch-in-r).

Reference

1. Ariey F, Witkowski B, Amaratunga C, Beghain J, Langlois AC, Khim N, et al. A molecular marker of artemisinin-resistant *Plasmodium falciparum* malaria. Nature. 2014;505:50–5. PubMed http://dx.doi.org/10.1038/nature12876

Technical Appendix Table 2. Data on the participants with parasites harboring mutation on K13 propeller gene

Mutation	Mutation	Time	Study site	Age, y	Sex
Non-synonymous	M442V	2012 Aug	Mfangano	12	M
	N554S	2012 Feb	Ungoye	7	F
	A569S	2013 Aug	Ungoye	13	F
	A578S	2012 Feb	Mfangano	16	F
				16	M
				17	M
				8	M
		2012 Aug	Mfangano	4	M
Synonymous	C439C	2012 Feb	Ungoye	9	M
				14	M
	S477S	2012 Feb	Takawiri	24	F
	Y500Y	2012 Aug	Mfangano	1	M
	N531N	2013 Aug	Ungoye	4	F
	G538G	2012 Feb	Mfangano	8	M
				16	F
				32	F
